# A bioinformatic analysis of T-cell epitope diversity in SARS-CoV-2 variants: association with COVID-19 clinical severity in the United States population

**DOI:** 10.3389/fimmu.2024.1357731

**Published:** 2024-05-09

**Authors:** Grace J. Kim, Jacob H. Elnaggar, Mallory Varnado, Amy K. Feehan, Darlene Tauzier, Rebecca Rose, Susanna L. Lamers, Maya Sevalia, Najah Nicholas, Elizabeth Gravois, Daniel Fort, Judy S. Crabtree, Lucio Miele

**Affiliations:** ^1^ Department of Genetics, Louisiana State University Health Sciences Center, New Orleans, LA, United States; ^2^ School of Medicine, Louisiana State University Health Sciences Center, New Orleans, LA, United States; ^3^ Department of Microbiology, Immunology, and Parasitology, Lousiana State University Health Sciences Center (LSUHSC), New Orleans, LA, United States; ^4^ Research and Development, Oschner Medical Center, New Orleans, LA, United States; ^5^ Department of Pathology, Louisiana State University Health Sciences Center, New Orleans, LA, United States; ^6^ Research and Development, BioInfoExperts, LLC, Thibodaux, LA, United States

**Keywords:** SARS-CoV-2, T cell epitope, COVID-19, bioinformatics, CD8 T cell epitope, HLA, vaccine design

## Abstract

Long-term immunity against severe acute respiratory syndrome coronavirus 2 (SARS-CoV-2) requires the identification of T-cell epitopes affecting host immunogenicity. In this computational study, we explored the CD8^+^ epitope diversity estimated in 27 of the most common HLA-A and HLA-B alleles, representing most of the United States population. Analysis of 16 SARS-CoV-2 variants [B.1, Alpha (B.1.1.7), five Delta (AY.100, AY.25, AY.3, AY.3.1, AY.44), and nine Omicron (BA.1, BA.1.1, BA.2, BA.4, BA.5, BQ.1, BQ.1.1, XBB.1, XBB.1.5)] in analyzed MHC class I alleles revealed that SARS-CoV-2 CD8^+^ epitope conservation was estimated at 87.6%–96.5% in spike (S), 92.5%–99.6% in membrane (M), and 94.6%–99% in nucleocapsid (N). As the virus mutated, an increasing proportion of S epitopes experienced reduced predicted binding affinity: 70% of Omicron BQ.1-XBB.1.5 S epitopes experienced decreased predicted binding, as compared with ~3% and ~15% in the earlier strains Delta AY.100–AY.44 and Omicron BA.1–BA.5, respectively. Additionally, we identified several novel candidate HLA alleles that may be more susceptible to severe disease, notably HLA-A*32:01, HLA-A*26:01, and HLA-B*53:01, and relatively protected from disease, such as HLA-A*31:01, HLA-B*40:01, HLA-B*44:03, and HLA-B*57:01. Our findings support the hypothesis that viral genetic variation affecting CD8 T-cell epitope immunogenicity contributes to determining the clinical severity of acute COVID-19. Achieving long-term COVID-19 immunity will require an understanding of the relationship between T cells, SARS-CoV-2 variants, and host MHC class I genetics. This project is one of the first to explore the SARS-CoV-2 CD8^+^ epitope diversity that putatively impacts much of the United States population.

## Graphical Abstract

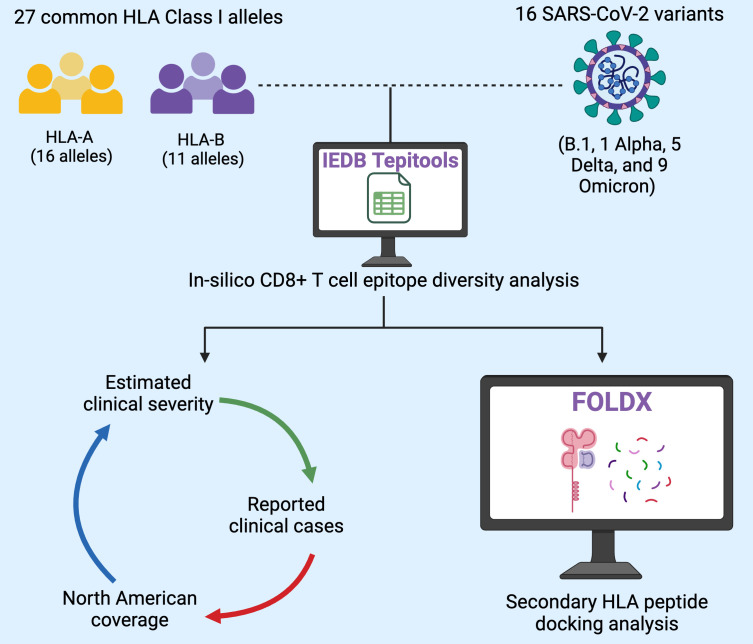

## 1 Introduction

Since the emergence of severe acute respiratory syndrome coronavirus 2 (SARS-CoV-2) in late 2019, the scientific community rapidly developed several therapeutic monoclonal antibodies and mRNA vaccines. Current vaccines elicit a short-lived humoral response against the SARS-CoV-2 spike protein, lasting an average of 3–4 months and requiring periodic boosters (1–3). Intriguingly, coronavirus disease 2019 (COVID-19) patients lacking humoral immune response due to treatment of hematological malignancies did not exhibit increased disease severity or mortality, suggesting that B-cell-mediated immunity may not be sufficient to confer long-term immunity against SARS-CoV-2 (4–6). In contrast, convalescent macaque models depleted of CD8^+^ T cells exhibited loss of host protection following reinfection, highlighting the importance of T-cell immunity in COVID-19 clinical presentation (7).

Cytotoxic CD8^+^ T cells are essential for the clearance of intracellular viral pathogens, such as SARS-CoV-2 (8–10). T-cell activation occurs through T-cell receptors binding to T-cell epitopes, described as peptide antigens bound by a human heterodimeric glycoprotein, known as a major histocompatibility complex (MHC). CD8^+^ T-cell antigen recognition is determined by MHC class I genes, which control antigenic peptide presentation on MHC class I molecules (11). Unlike the invariant β_2_-microglobulin subunit, the α subunit of MHC class I proteins is highly polymorphic, with the most polymorphic genes being human leukocyte antigens (*HLA*) *HLA-A*, *HLA*-*B*, and *HLA*-*C*; these subunits have an estimated 1,939, 2,577, and 1,595 allotypes, respectively (11, 12). Therefore, the considerable individual diversity generated from HLA polymorphism is a proposed explanation for the differential clinical severity of COVID-19 variants seen between individuals, since the epitope repertoire from one patient is likely to be substantially different from the next (13–15). Select studies have sequenced the *HLA* alleles and SARS-CoV-2 T-cell epitopes of convalescent patients (4, 15–18). However, current research on T-cell response to COVID-19, especially analysis exploring the relationship between *HLA* molecules and viral CD8^+^ epitopes on a population/epidemiological level, remains limited. Previously published research has already identified several HLA alleles associated with increased (HLA-A*25:01, HLA-B*46:01, and *HLA*-B*27:07) or decreased (HLA-B*07:02, HLA-B*15:03, and *HLA*-B*51:01) clinical severity in convalescent patients (**Table 1**) (13, 19–21), but none have explored the entire epitope repertoire of variants of concern (VOC) gene products in the most common HLA allotypes.

**Table 1:** Estimated ΔG for SARS-CoV-2 CD8+ peptides docked with HLA-B*15:01 by FOLDX.

Peptide	Covid Strain	Mutation Type	Wuhan Predicted Binding	VOC Predicted Binding	ΔG (kcal/mol)		Notes (Altered from X to Y)
					8elg	3c9n	
ALPFNDGVY	XBB.1.5	Spike Increased Binding	0.76	0.59	-0.0995896	1.60678	VLPFNDGVY to ALPFNDGVY
LERDLPQGF	XBB.1.5	Spike Decreased Affinity	0.12	0.82	0.445307	3.22076	LVRDLPQGF to LERDLPQGF
GQTGNIADY	XBB.1.5	Spike Decreased Affinity	0.08	0.18	-2.00747	1.14488	GQTGKIADY to GQTGNIADY
HQPYRVVVL	XBB.1.5	Spike Increased Binding	0.59	0.56	0.91709	-3.04112	YQPYRVVVL to HQPYRVVVL
LVKQLSSKF	XBB.1.5	Spike Increased Binding	0.06	0.04	-1.11521	-0.832341	LVKQLSSNF to LVKQLSSKF
CVADYSVIY	XBB.1.5	Spike Increased Binding	0.36	0.31	1.03023	0.182606	CVADYSVLY to CVADYSVIY
NCYSPLQSY	XBB.1.5	Spike Increased Binding	0.7	0.42	1.82501	0.759358	NCYFPLQSY to NCYSPLQSY
KLDDKGPNF	BA.1.1	Nucleocapsid Increased Binding	1	0.5	-0.580903	-1.76596	KLDDKDPNF to KLDDKGPNF

Predicted binding values reflect predicted consensus percentile ranks generated from IEDB’s Tepitools, as described in the methods. Low scores correspond to high predicted binding affinities.

Highlighted letters indicate amino acid alterations from the original Wuhan sequence to the respective SARS-CoV-2 strain.

SARS-CoV-2 VOC and subvariants accumulate mutations in the genes for their protein products, such as spike, membrane, and nucleocapsid proteins, potentially affecting the binding affinity and immunogenicity of T-cell epitopes. These mutations and the resulting alterations to MHC-I binding affinity may influence COVID-19 clinical characteristics, such as viral transmissibility, protection against neutralizing antibodies, risks of reinfection, and disease severity (15, 22, 23), as well as the risk of post-acute sequelae of COVID-19 infection (PASC or long COVID) (24–26). Given that an estimated 3% of CD8^+^ T-cell epitopes are affected by mutations conferred in various VOCs, certain HLA alleles may have more (or less) propensity to be strongly affected by mutations in specific VOCs (15).

This manuscript distinguishes SARS-CoV-2 variant-specific CD8^+^ T-cell epitopes of spike, membrane, and nucleocapsid gene products for 27 of the most frequent *HLA-A* and *HLA*-*B* alleles. The purpose of this computational study was to model the immunogenic effects and clinical severity of SARS-CoV-2 variants in the most common MHC class I alleles in the United States population. Our bioinformatics approach integrates the use of Ensembl’s COVID-19 genome browser, Immune Epitope Database and Analysis Resource tool TepiTool, and ExPASy translate tool (27–29).

## 2 Materials and methods

### 2.1 SARS-CoV-2 viral genome sequencing

Specimens were received by the LSUHSC Precision Medicine Laboratory from various collection sites representing the Louisiana patient population for public health screening purposes. RNA extraction was performed using the Zymo Quick DNA/RNA Viral MagBead kit automated on a Tecan Fluent liquid handling workstation. The resulting viral RNA was used for library generation and next-generation sequencing using the Illumina COVID-Seq workflow as per the manufacturer’s instructions. Libraries were pooled (up to 192 samples/run) and loaded on an Illumina NextSeq550Dx in RUO mode, with 74 cycles of paired-end sequencing using a 150-cycle mid output reagent cartridge and flow cell. Initial data processing and QC was performed using the DRAGEN COVID-Seq Test (EUA) v.1.2.2 application on the cloud-based BaseSpace sequence analysis hub hosted by Illumina. BaseSpace project share links were provided to BioInfoExperts, LLC for sequence processing and analysis in FoxSeq software (www.foxseqllc.com). Briefly, sequences were quality-filtered using Trimmomatic (30) and mapped to the reference using Bowtie2 (31). Variant calling and consensus sequence generation were performed using bcftools (32). Nucleotides at any position were only assigned if the sequencing depth was >200 and the allele frequency was 80%. Lineages were assigned using pangolin (https://cov-lineages.org). Consensus sequences were uploaded to GISAID and NCBI SARS-CoV-2 viral genome data repositories.

### 2.2 SARS-CoV-2 variant sequence comparison and protein peptide sequence generation

Genome sequences of SARS-CoV-2 variants were blasted against the originally sequenced Wuhan strain (INSDC accession CGA_009858895.3) using Ensembl’s (RRID: SCR_002344) SARS-CoV-2 genome browser (29). Variant-specific cDNA sequences for transcripts were generated from Ensembl’s SARS-CoV-2 genome browser (RRID: SCR_024704). SARS-CoV-2 variant-specific cDNA for spike, membrane, and nucleocapsid was converted to amino acid (protein) sequences, using the ExPASy translate tool (RRID: SCR_024703) (27).

### 2.3 TepiTool IEDB analysis of coronavirus T-cell epitopes

The prediction of MHC-I epitope binding to variant-specific S, M, and N was generated through the Immune Epitope Database and Analysis Resource (IEDB) (RRID: SCR_006604), via TepiTool utilizing the IEDB-recommended default prediction (33). Spike, membrane, and nucleocapsid were selected because, for the most part, spontaneous CD8^+^ responses against SARS-CoV-2 T-cell epitopes target the proteins they encode (16). A panel of 27 most frequent A and B alleles was used for MHC-I epitope binding analysis. The specific alleles included were as follows: HLA-A*01:01, HLA-A*02:01, HLA-A*02:03, HLA-A*02:06, HLA-A*03:01, HLA-A*11:01, HLA-A*23:01, HLA-A*24:02, HLA-A*26:01, HLA-A*30:01, HLA-A*30:02, HLA-A*31:01, HLA-A*32:01, HLA-A*33:01, HLA-A*68:01, HLA-A*68:02, HLA-B*07:02, HLA-B*08:01, HLA-B*15:01, HLA-B*35:01, HLA-B*40:01, HLA-B*44:02, HLA-B*44:03, HLA-B*51:01, HLA-B*53:01, HLA-B*57:01, and HLA-B*58:01. IEDB’s default prediction method reflects consensus across ANN, SMM, and CombLib predictors and was used to select peptides with predicted consensus percentile ranks ≤1 (28). Low scores correspond to high predicted affinities.

### 2.4 FoldX peptide docking of HLA-B*15:01

Molecular docking was adapted from Mazumder et al. (34). The RepairPDB method from FoldX (RRID: SCR_008522) Suite 5.0 was initially used to repair the structures obtained from the RCSB Protein Data Bank (**Supplementary Table S1**) (35). This allows for the use of the structures in downstream FoldX tools. BuildModel was used to convert from the peptide in the structure to the original SARS-COV-2 CD8^+^ peptide. BuildModel was used again to convert from the original SARS-COV-2 CD8^+^ peptide to the mutated peptide. The estimated ∆*G* (kcal/mol) was then used to create a heatmap in R (v4.2.1) with the ComplexHeatmap function (36). Python scripts used to run FoldX can be found at github.com/elnaggarj/FoldX-PeptideDocking.

## 3 Results

### 3.1 Spike, membrane, and nucleocapsid nucleotide alterations between one pre-Alpha, one Alpha, five Delta, and nine Omicron SARS-CoV-2 variants over time

**Figure 1:**
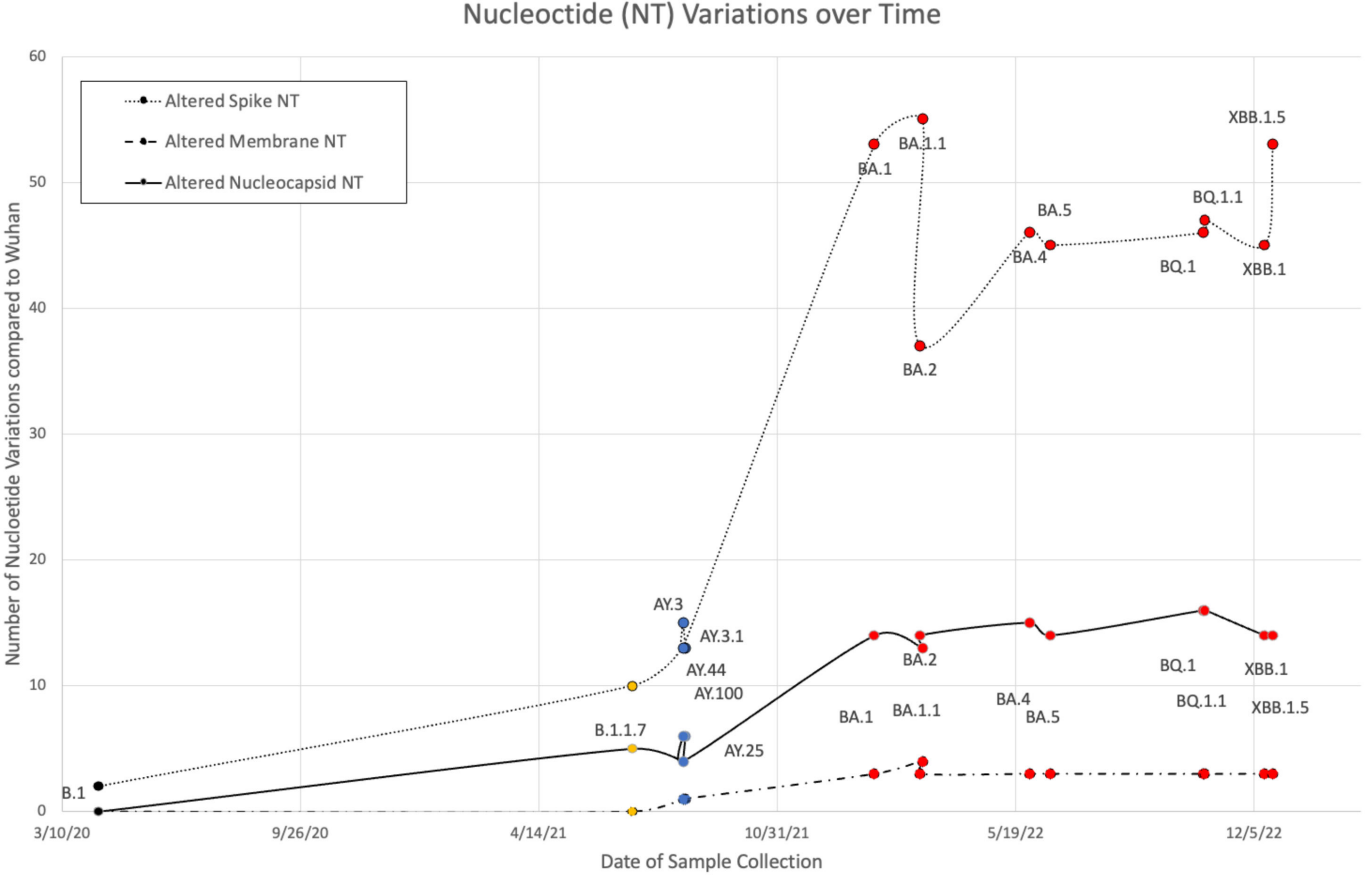
Nucleotide (NT) variations of spike, membrane, and nucleocapsid over time between B.1 (colored black), Alpha (orange), five Delta (blue), and nine Omicron (red) variants when compared against the original Wuhan strain.

B.1, Alpha (B.1 and B.1.1.7), five Delta (AY.100, AY.25, AY.3, AY.3.1, and AY.44), and nine Omicron (BA.1, BA.1.1, BA.2, BA.4, BA.5, BQ.1, BQ.1.1, XBB.1, and XBB.1.5) VOCs were sequenced from the Louisiana patient population between 9 April 2020 and January 2023. Variant FASTAs were compared with the ancestral Wuhan strain (NCBI: NC_045512.2) using BLAST to determine nucleotide differences (**Figure 1**). Alpha and Delta strains displayed minimal variance, with Delta exhibiting 12–15, 1, and 4–6 nucleotide (NT) variations in S, M, and N, respectively. B.1 and B.1.1.7 showed alterations in spike (2 NT in B.1; 10 NT in B.1.1.7) and nucleocapsid (5 NT in B.1.1.7) although M remained identical to the original Wuhan strain. In comparison, Omicron variants exhibited 37–55, 3–4, and 13–16 NT variations in S, M, and N, respectively (**Figure 1**). Among the three protein products analyzed, membrane and nucleocapsid sequences were highly conserved, with M experiencing only 0–4 NT changes between the 16 variants analyzed (M: 665–669/669 NT = 99.4%–100% conservation; N: 1,244–1,256/1,260 = 98.7%–99.8%; S: 3,830–3,776/3,831 = 98.5%–99.9%). Additionally, there was limited mutational divergence seen in Omicron variants between March 2022 and December 2022, suggesting a possible plateau in genetic drift within the Omicron family of SARS-CoV-2.

### 3.2 Epitope differences between 16 variants of spike, membrane, and nucleocapsid when compared against the ancestral Wuhan strain

We generated predictive estimates of MHC-I epitopes to variant-specific S, M, and N using the IEDB Resource TepiTool, utilizing the IEDB-recommended default prediction. A panel of 27 most frequent A and B alleles were used for MHC-I epitope binding analysis, which encompassed 16 HLA-A (HLA-A*01:01, HLA-A*02:01, HLA-A*02:03, HLA-A*02:06, HLA-A*03:01, HLA-A*11:01, HLA-A*23:01, HLA-A*24:02, HLA-A*26:01, HLA-A*30:01, HLA-A*30:02, HLA-A*31:01, HLA-A*32:01, HLA-A*33:01, HLA-A*68:01, and HLA-A*68:02) and 11 HLA-B (HLA-B*07:02, HLA-B*08:01, HLA-B*15:01, HLA-B*35:01, HLA-B*40:01, HLA-B*44:02, HLA-B*44:03, HLA-B*51:01, HLA-B*53:01, HLA-B*57:01, and HLA-B*58:01) alleles. Utilizing the haplotype frequency estimates provided by the National Marrow Donor Program (37), the 16 HLA-A alleles make up 92.4% of the population in Caucasians, 69.2% in African Americans, 74% in Asian, and 83% in Hispanics. Similarly, the 11 HLA-B alleles represent 67.7% of Caucasians, 44.8% of African Americans, 39.2% of Asians, and 39.8% of Hispanics. CD8^+^ epitope repertoires, comprising the 27 most common HLA-A and HLA-B alleles, were generated for 16 SARS-CoV-2 variants and the ancestral Wuhan strain. The original S, M, and N protein products resulted in a repertoire of 1,081, 237, and 289 predicted CD8^+^ epitopes, respectively. From the 16 SARS-CoV-2 variant spike proteins, we identified a range of 1,077–1,115 CD8^+^ T-cell epitopes. Variant-specific membrane epitopes ranged between 236 and 241, with nucleocapsid CD8^+^ repertoires comprising 289–298 epitopes for the 27 HLA alleles analyzed.

**Figure 2:**
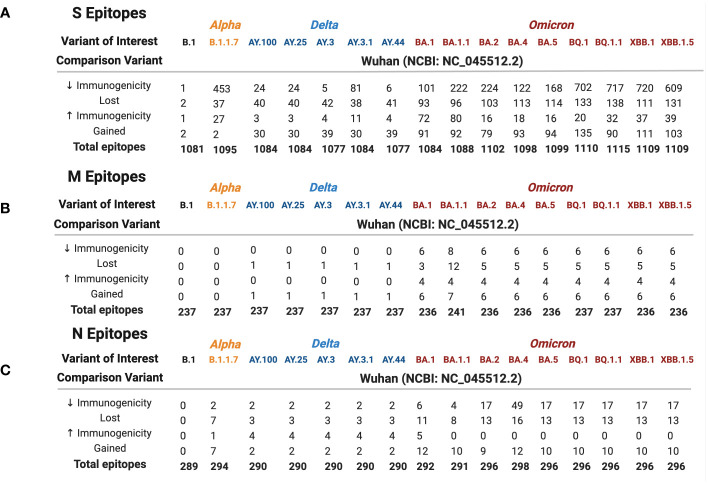
Spike **(A)**, membrane **(B)**, and nucleocapsid **(C)** epitope differences between variants of interest (B.1 labeled black, Alpha in orange, Delta in blue, and Omicron in red) when compared against the ancestral Wuhan strain. Predicted binding of SARS-CoV-2 S, M, and N epitopes was generated using the IEDB database TepiTool for the 27 most common HLA-A and HLA-B alleles.

**Figure 3:**
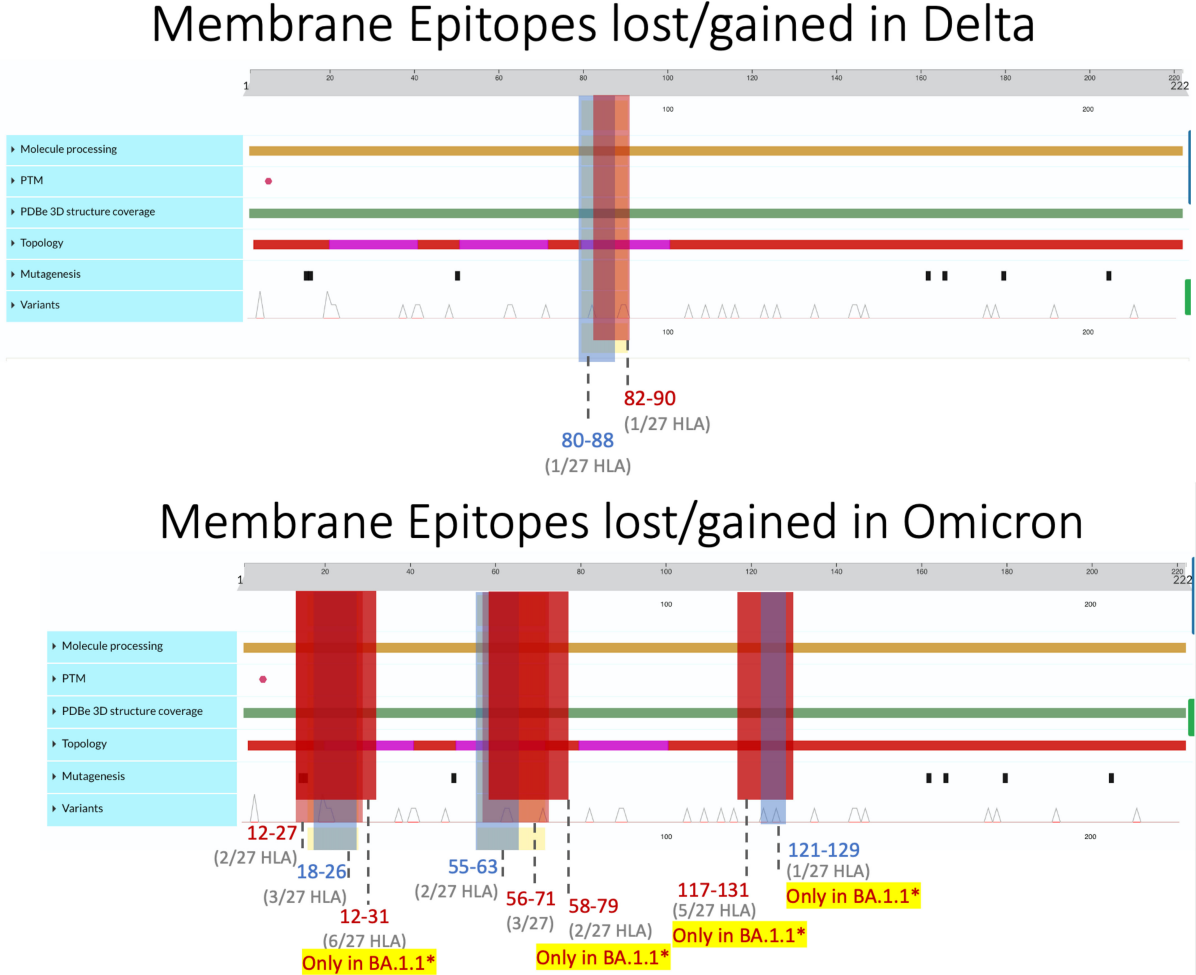
Membrane epitopes lost (regions colored red) and gained (colored blue) in Delta (top) and Omicron (bottom) when compared against the ancestral Wuhan strain. Protein characteristics were generated using UniProt’s Feature Viewer (38).

Wuhan S, M, and N repertoires were compared against 16 variants (B.1, Alpha, five Delta, and nine Omicron) to identify epitopes that were lost, gained, or altered in estimated HLA binding affinity between variants (**Figure 2**). In general, a balanced number of epitopes were lost and gained for all variants; however, BA.1.1 M, BA.4 N, B.1.1.7 S, and BQ.1.1 S repertoires sustained greater epitope loss than gain (**Figures 2A–C**, **3**, *bottom*), which may contribute to explaining the increased transmission and breakthrough cases seen in these subvariants (39, 40). Additionally, spike epitopes in the early variants (B.1 and B.1.1.7) and Omicron VOCs experienced a greater number of epitopes predicted to have reduced binding affinity than increased affinity.

#### 3.2.1 Spike epitopes were the least conserved, compared with membrane and nucleocapsid

**Figure 4:**
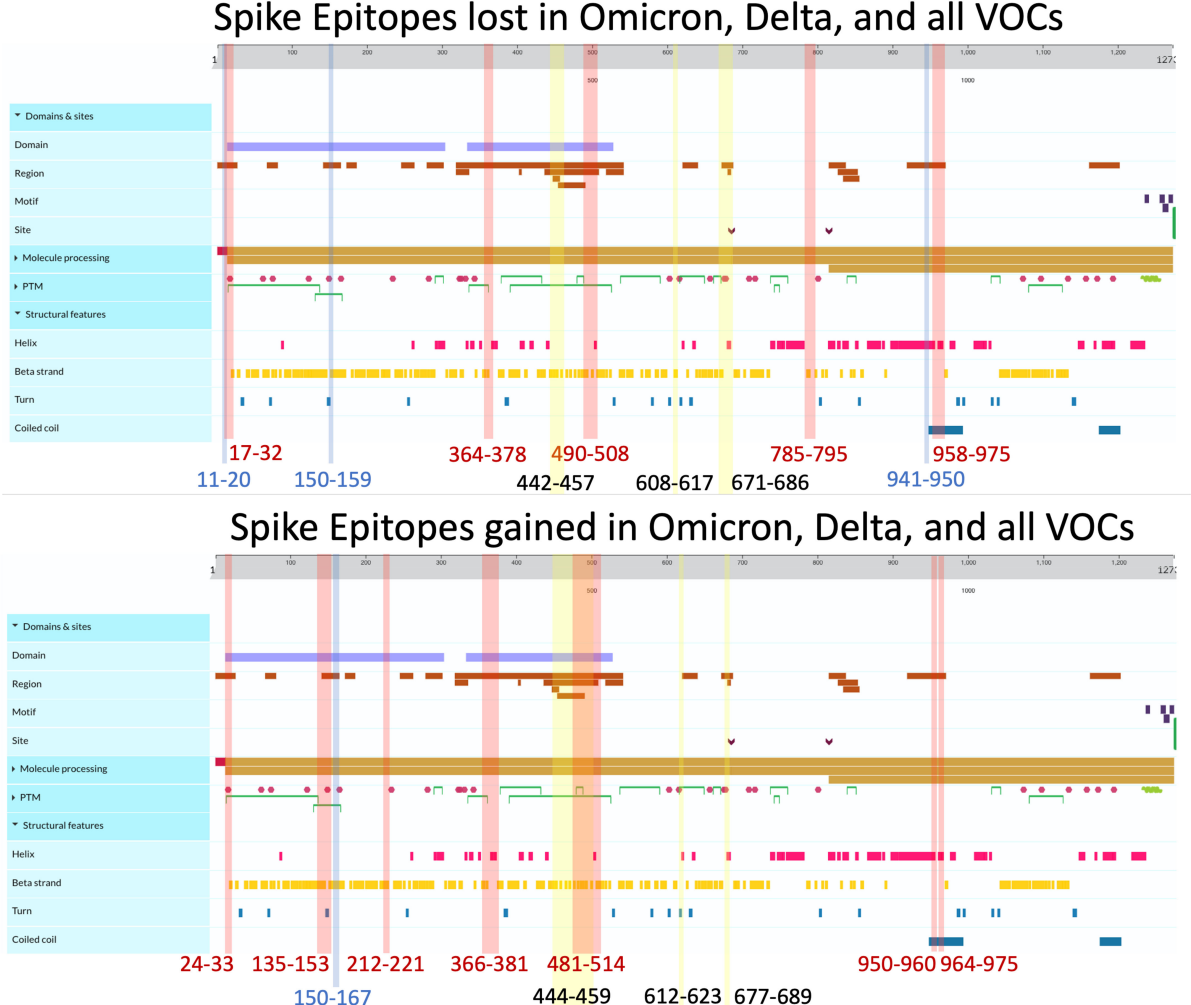
Spike epitopes gained (top) and lost (bottom) when compared against the ancestral Wuhan strain. Colored regions and numbers refer to amino acid locations of predicted epitope alterations, with red indicating changes seen in Omicron, blue for Delta, and yellow for epitopes affected in all 16 variants. Protein characteristics were generated using UniProt’s Feature Viewer (38).

Among the three viral proteins we examined, spike epitopes were least conserved, with S, M, and N epitopes experiencing 87.6%–99.8%, 92.5%–100%, and 94.6%–100% conservation, respectively. Across all the variants studied, Omicron BQ.1.1 S epitopes experienced the most loss, with 138/1,115 = 12.4% affected, while strain B.1 only lost 2 epitopes out of 1,081 total (0.0019%) compared with the original Wuhan strain. Among the 14 Delta and Omicron spike proteins, the largest area of conservation, defined as a region experiencing no epitope loss or gain, was found between amino acids (AA) 987–1,205 within the 1273 AA protein (**Figure 4**). As seen in the two other protein products, S epitopes that were lost were generally replaced by alternate epitopes that were gained in the same regions. However, all Delta variants lost two epitopes (VSSQCNLR and SQCVNLRTR), affecting HLA-A*31:01 and HLA-A*68:01, without experiencing epitope gains (**Supplementary Figure 2**). These epitopes spanned the AA 11–21 region, affecting the tail end of the hydrophobic signal peptide and the S1 subunit in the S protein.

As shown in **Figure 2A**, 41.4% of B.1.1.7 (Alpha VOC) spike epitopes were estimated to have reduced immunogenicity, while only 2.5% (27/1,092 epitopes) demonstrated increased predicted HLA binding. As SARS-CoV-2 mutated, an increasing proportion of epitopes were predicted to have reduced HLA binding, with 70% of Omicron BQ.1–XBB.1.5 S epitope repertoires experiencing decreased predicted binding affinity (as compared with the roughly 3% and 15% affected in Delta AY.100–AY.44 and Omicron BA.1–BA.5 variants, respectively) (**Figures 1**, **2A**). When compared with the ancestral Wuhan spike, XBB.1 S epitopes experienced the greatest decrease in predicted immunogenicity, with 64.9% (720/1,109 epitopes; **Figure 2A**) of its CD8- T-cell repertoire demonstrating a reduction in estimated binding affinity, while only 37 epitopes (3.3%) were estimated to have increased HLA binding. Additionally, all 27 *HLA-A* and *HLA*-*B* alleles had decreased predicted binding affinity for B.1.1.7 and BA.1–XBB.1.5 spike epitopes.

#### 3.2.2 Membrane epitopes were most conserved with balanced gain and loss maintained in all variants

Membrane epitopes sustained minimal alterations, with BA.1.1 losing the most (18/241 = 7.5%) and AY.100–AY.44 losing the least (1/237 = 0.04%) epitopes between Delta and Omicron variants (**Figures 1**, **2B**). Alpha membrane epitopes were conserved unaltered from the original Wuhan variant sequenced. In general, M epitope loss was accompanied by balanced epitope gains across all VOCs, with similar patterns seen between epitopes with altered predicted binding affinity (**Figure 2A**). For all Delta variants, HLA-A*68:02 lost the ability to bind epitope TAMACLVGL, while HLA-B*51:01 gained IAIAMCLV between AA 80 and 90. Likewise, for the nine Omicron variants, two membrane segments (AA 12–27 and AA 55–71) experienced balanced epitope loss and gain (**Figure 3**).

BA.1.1 M protein lost significantly more epitopes than the other variants, affecting 15/27 HLA alleles, while the other 8 Omicron variants sustained epitope loss in only 5 HLA alleles (**Figures 2B**, **3**, *bottom*). Additionally, BA.1.1 M contained a third region between 117 and 129 AA wherein HLA-A*03:01, *HLA*-A*26:01, HLA-A*30:02, HLA-A*31:01, and HLA-A*33:01 endured epitope loss of NILLNVPLY and PLYGTILTR (**Figure 3**, *bottom*). Unlike other regions, only HLA-B*08:01 gained an epitope within the AA 117–129 segment. The region between AA 132 and 222 was found to be conserved, with no predicted epitopes being lost or gained.

#### 3.2.3 Unbalanced nucleocapsid epitope gain/loss and alterations in predicted binding, with more epitopes experiencing decreased predicted binding

**Figure 5:**
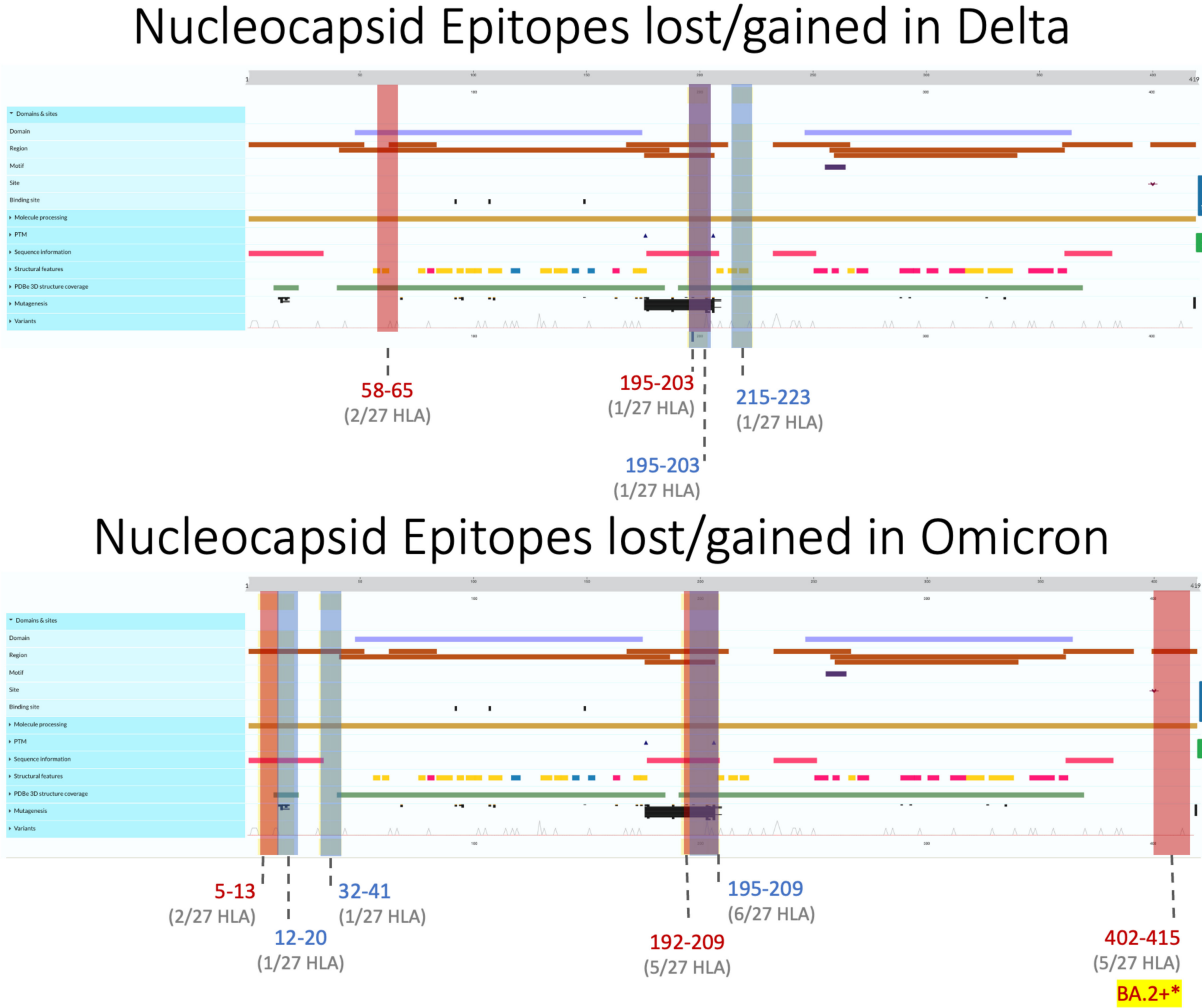
Nucleocapsid epitopes lost (regions colored red) and gained (in blue) in Delta (top) and Omicron (bottom) variants when compared against the ancestral Wuhan strain. Protein characteristics were generated using UniProt’s Feature Viewer.

Like M, N epitopes were highly conserved, with the greatest loss seen in BA.4 (16/298 = 5.4%) and conservation in AY.100/AY.25/AY.44 (3/290 = 1%) among Omicron and Delta variants. N epitopes experienced no loss or gain between AA 66–194 and AA 237–401 in all VOCs. Although nucleocapsid epitopes experienced *numerically* balanced gain and loss across VOCs (**Figure 2C**), further analysis revealed AA 192–209 to be the only region where epitopes were both gained and lost, including the Alpha variant (**Figure 5**). Within this region, more *HLAs* sustained gain/loss in Omicron (6/27 *HLA* gain; 5/27 *HLA* loss) and Alpha (6/27 HLA gain; 5/27 HLA loss) VOCs than Delta VOCs (1/27 *HLA* gain; 1/27 *HLA* loss) in this region. Unlike the other two SARS-CoV-2 VOC families, Alpha only experienced epitope loss/gain between the AA 195 and 237 region, wherein epitope SSRGTSPAR was gained in *HLA-A:03:01*, HLA-A*11:01, *HLA*-A*30:01, *HLA*-A*31:01, *HLA*-*A:33:01*, and *HLA*-*A:68:01*, while RNSTPGSSK and NSTPGSSKR were lost in HLA-A*03:01, *HLA*-A*11:01, *HLA*-A*30:01, *HLA*-A*33:01, and *HLA*-A*68:01.

Across all Delta N epitope repertoires, HLA-A*23:01 and HLA-A*24:02 lost the ability to bind to QHGKEGLKF between 58 and 65 AA, while HLA-B*40:01 gained binding to GDAALALLL in AA 215–223 (**Figure 4**, *top*). Between the nine Omicron variants, HLA-B*07:02 gained APTRITFGGP epitope binding between 12 and 20 AA, while HLA-A*31:01 gained two epitopes (RSGARSKQR and SGARSKQRR) between AA 32 and 41. Omicron-specific N epitope loss was found between AA 5 and 13, where HLA-B*07:02 and HLA-B*08:01 lost the ability to bind to GPQNQRNAL. In addition, SSRGTSPAR (AA 402–415) loss was found in BA.2–XBB.1.5 VOCs for 5/27 alleles: HLA-A*03:01, HLA-A*11:01, HLA-A*30:01, HLA-A*33:01, and HLA-A*68:01. Omicron VOCs BA.1.1–XBB.1.5 sustained decreased predicted binding affinity in epitopes [4 epitopes (BA.1.1 N)–49 epitopes (BA.4 N)], with zero epitopes gaining predicted binding (**Figure 2C**). BA.4 had 40 unique peptides affecting 16/16 *HLA-A* and 8/11 *HLA-B* alleles (**Supplementary Figure 1**).

#### 3.2.4 Gained epitopes conserved in Omicron and Delta variants

**Figure 6:**
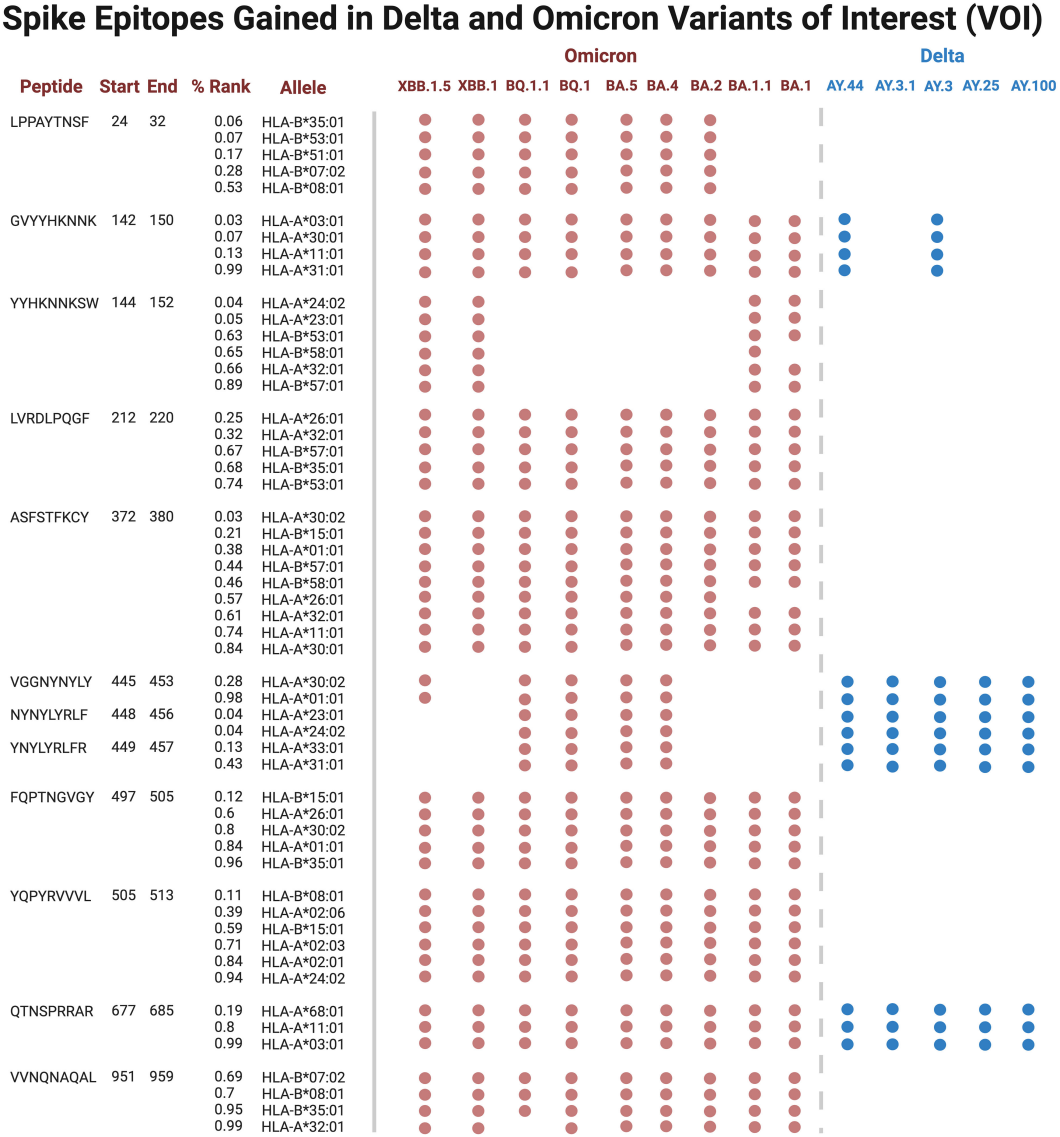
Spike epitopes gained in nine Omicron and five Delta variants, when compared against the original Wuhan strain. Figures were generated using BioRender (RRID: SCR_018361).

**Figure 7:**
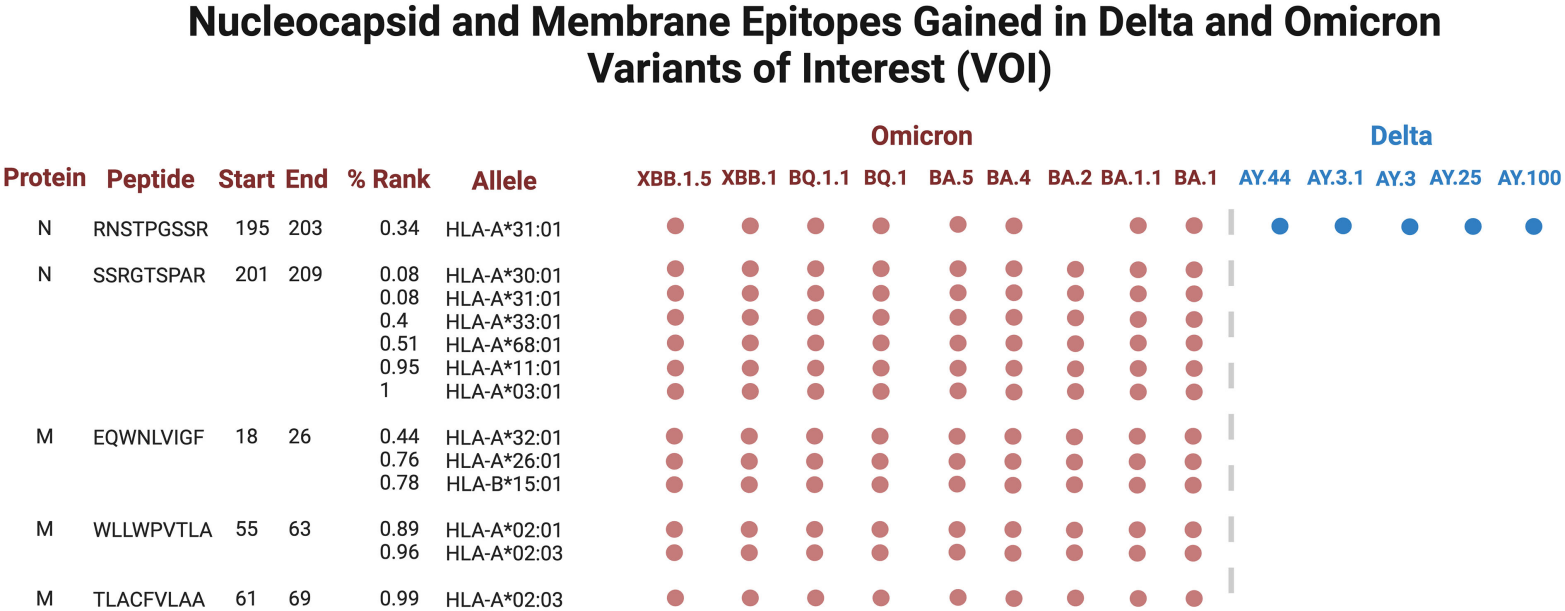
Nucleocapsid (N) and membrane (M) epitopes gained in nine Omicron (colored red) and five Delta (blue) variants when compared against the original Wuhan strain. Figures were generated using BioRender (RRID: SCR_018361).

Several epitopes were gained or conserved across Delta and Omicron families, including the spike epitopes, GVYYHKNNK, QTNSPRRAR, VGGNYNYLY, NYNYLYRLF, and YNYLYRLFR, as well as the nucleocapsid epitope RNSTPGSSR (**Figures 6**, **7**). Of the gained S epitopes, ASFSTFKCY encompassed the greatest number of HLAs analyzed (9/27), estimated to affect 6/16 *HLA*-*A* encompassing 31.2% of the population in Caucasian American (EUR), 22.2% of African American (AFA), 30.3% of Asian American and Pacific Islander (API), and 21.8% of Hispanic and Latino Americans (HIS), and 3/11 *HLA-B* alleles (11% EUR, 5% AFA, 11.3% API, and 5.5% HIS). Likewise, the nucleocapsid epitope SSRGTSPAR was gained in 6/16 *HLA-A* alleles, comprising of 27.2% EUR, 23.5% AFA, 27.8% API, and 26.1% HIS population in the United States (**Figure 7**).

### 3.3 Secondary *in-silico* structural epitope binding using FoldX

**Figure 8:**
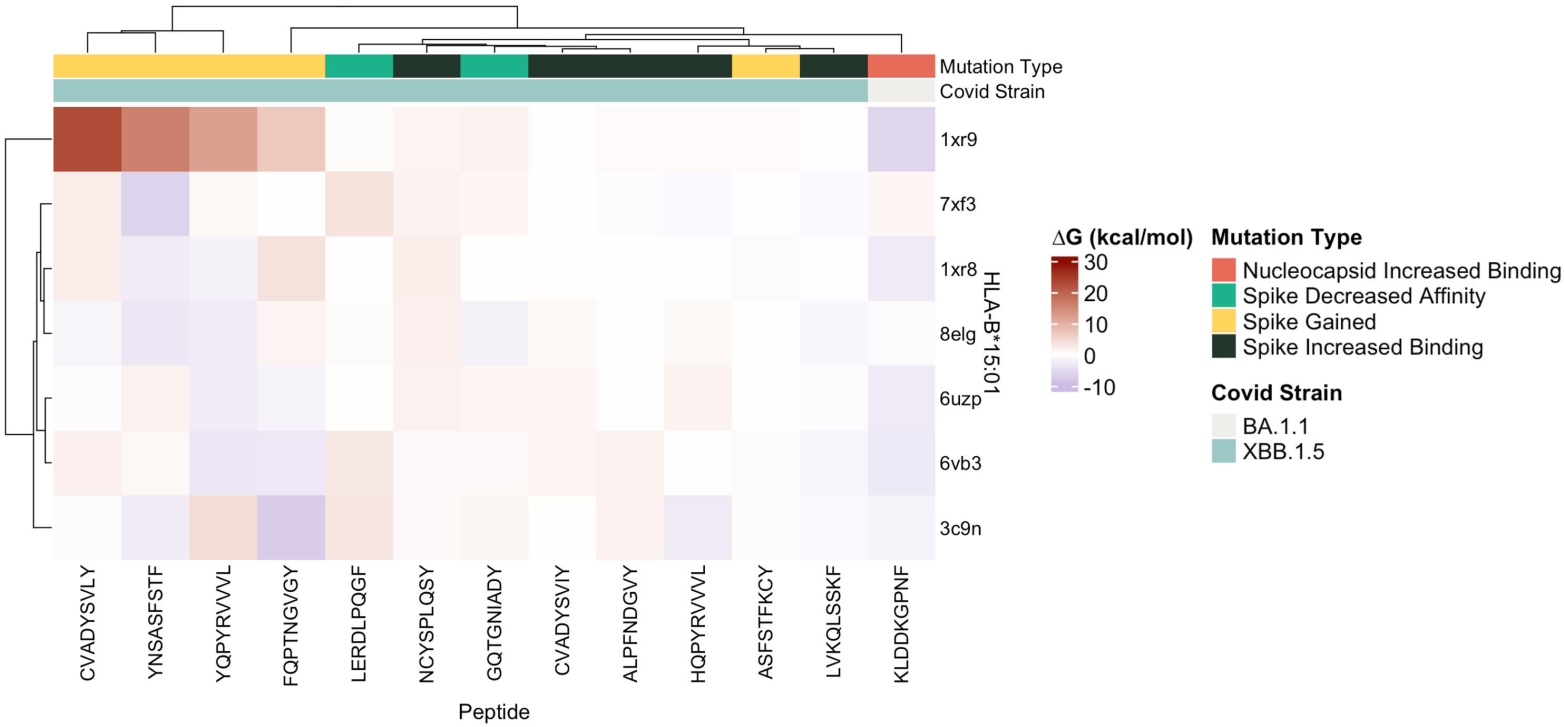
Heatmap of estimated ΔG (kcal/mol) values predicted for ligands docked with target SARS-CoV-2 epitopes with crystalized HLA-B*15:01 structures (*n* = 7) by FoldX.

Protein-peptide binding free energy of SARS-CoV-2 peptides and HLA-B*15:01 (*n* = 7, **Supplementary Table 1**) was computationally determined using FoldX (**Tables 1**, **2**, **Supplementary Tables 1–3**). HLA-B*15:01 was selected because the allele is both common and has been the focus of recent publications (**Tables 3**, **4**) (47–49). Of the seven HLA-B*15:01 structures downloaded from the Protein Data Bank, only one structure, 8ELG, was complexed with SARS-CoV-2 epitopes (**Supplementary Table 1**). Protein-peptide binding analysis for all seven HLA-B*15:01 structures returned a 58% match between FoldX-generated binding free energy/ΔG and IEDB-predicted consensus percentile ranks. The predicted binding match rate jumped to 64% in HLA-B*15:01 complexed with *Coronaviridae* peptides 8ELG and 3C9N (**Tables 1**, **2**, **Supplementary Table 1**). The nucleocapsid peptide KLDDKGPNF, which had mutated from KLDDKDPNF (Wuhan) to KLDDKGPNF (BA.1.1), exhibited the greatest match rate (85.7% = 6/7; **Figure 8**, **Supplementary Table 2**).

**Table 2:** Estimated binding energy for SARS-CoV-2 CD8+ peptides docked with HLA-B*15:01 by FOLDX.

Peptide	Covid Strain	Mutation Type	VOC Predicted Binding	ΔG (kcal/mol)	
				8elg	3c9n
CVADYSVLY	XBB.1.5	Spike Gained	0.36	-1.63154	-0.598217
YNSASFSTF	XBB.1.5	Spike Gained	0.96	-3.72574	-2.75697
ASFSTFKCY	XBB.1.5	Spike Gained	0.21	-0.175898	0.447979
FQPTNGVGY	XBB.1.5	Spike Gained	0.12	1.41429	-7.62166
YQPYRVVVL	XBB.1.5	Spike Gained	0.59	-3.1373	4.41745
CVADYSVLY	XBB.1.5	Spike Gained	0.36	-1.63154	-0.598217
YNSASFSTF	XBB.1.5	Spike Gained	0.96	-3.72574	-2.75697
ASFSTFKCY	XBB.1.5	Spike Gained	0.21	-0.175898	0.447979

Predicted binding values reflect predicted consensus percentile ranks generated from IEDB’s Tepitools, as described in the methods. Low scores correspond to high predicted binding affinities.

**Table 3:** Summary of *HLA* haplotype United States population frequencies and clinical associations.

European American ancestry frequency	European frequency rank	African American ancestry frequency	African American frequency rank	Asian American (AAPI) frequency rank	AAPI frequency rank	Hispanic and Latino American frequency	Hispanic and Latino American frequency rank	Allele		*X_total_*	COVID-19 induced clinical associations
0.0313	7	0.0141	21	0.0130	18	0.0271	13	HLA-A*32:01	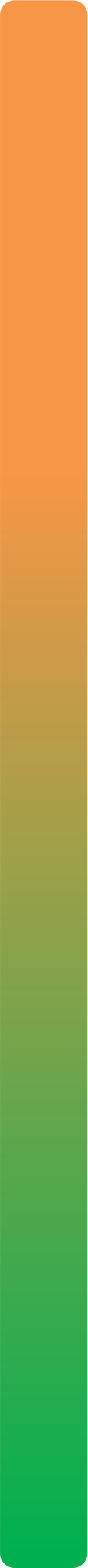	-62	
0.0295	8	0.0141	20	0.0390	8	0.0289	11	HLA-A*26:01		-55	
0.0092	15	0.0622	6	0.0006	40	0.0281	12	HLA-A*30:02		-55	Infection in USA
0.0134	13	0.0691	4	0.0206	12	0.0211	15	HLA-A*30:01		-48	
0.0869	4	0.0221	15	0.1824	1	0.1232	2	HLA-A*24:02		-46	Associated with autoimmunity*
0.0168	12	0.1077	2	0.0023	27	0.0369	10	HLA-A*23:01		-42	
0.0099	14	0.0212	16	0.0011	35	0.0196	16	HLA-A*33:01		-36	
0.0020	21	0.0002	56	0.0483	6	0.0392	9	HLA-A*02:06		-34	
0.0250	9	0.0368	11	0.0186	13	0.0469	6	HLA-A*68:01		-26	
0.0000	NA	0.0002	48	0.0316	10	0.0003	59	HLA-A*02:03		-25	
0.0564	5	0.0158	18	0.1790	2	0.0462	7	HLA-A*11:01		-19	Associated with autoimmune* and severe disease
0.1435	3	0.0813	3	0.0260	11	0.0791	3	HLA-A*03:01		-18	Protected in Russia
0.1718	2	0.0474	8	0.0508	5	0.0670	4	HLA-A*01:01		-17	Severe infection in Russia
0.2960	1	0.1246	1	0.0946	3	0.1940	1	HLA-A*02:01		-17	Protected in Russia
0.0235	10	0.0104	22	0.0325	9	0.0479	5	HLA-A*31:01		-13	
0.0085	16	0.0651	5	0.0003	46	0.0246	14	HLA-A*68:02		-13	Associated with reduced risk of ICU admittance
**0.9237**		**0.6924**		**0.7405**		**0.8301**		**Sum of HLA-A frequencies**			
0.0454	9	0.0218	16	0.0628	2	0.0578	3	HLA-B*51:01	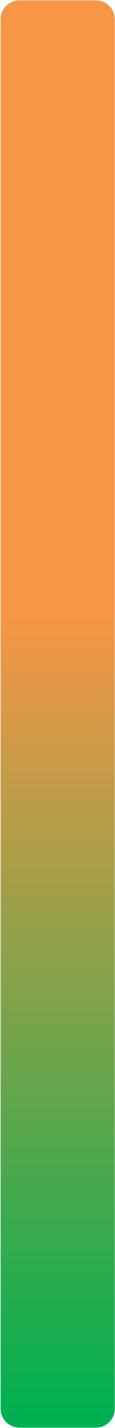	-40	Associated with severe disease
0.1253	2	0.0384	9	0.0164	21	0.0445	6	HLA-B*08:01		-39	Associated with autoimmunity*
0.0571	5	0.0649	3	0.0427	5	0.0635	1	HLA-B*35:01		-37	Associated with Subacute thyroiditis
0.0032	32	0.1125	1	0.0009	66	0.0155	21	HLA-B*53:01		-34	
0.0047	27	0.0351	11	0.0577	4	0.0145	23	HLA-B*58:01		-32	
0.1399	1	0.0730	2	0.0263	15	0.0545	4	HLA-B*07:02		-29	Associated with severe disease
0.0901	3	0.0212	17	0.0076	32	0.0333	9	HLA-B*44:02		-29	
0.0496	7	0.0537	6	0.0424	6	0.0608	2	HLA-B*44:03		-18	
0.0665	4	0.0098	23	0.0348	11	0.0288	10	HLA-B*15:01		-17	Survival in Egypt, Asymptomatic in USA
0.0383	10	0.0048	35	0.0207	18	0.0118	29	HLA-B*57:01		-17	
0.0564	6	0.0133	21	0.0798	1	0.0135	26	HLA-B*40:01		-12	
**0.6767**		**0.4483**		**0.3922**		**0.3985**		**Sum of HLA-B frequencies**			

Summary of allelic frequencies and clinical associations for the 27 HLA-A and HLA-B analyzed. **X**_**total**_ describes HLA-predicted clinical severity with more negative values indicating greater predicted clinical severity (Equation 2). Allelic frequencies were adapted from Gragert et al. (37).

*Autoimmunity reflects new-onset autoimmune symptoms following COVID-19 infection. Bolded numbers indicate estimated population coverage of the HLA-A (top) and HLA-B (bottom) alleles analyzed in this study.

**Table 4:** Summary of *HLA* CD8+ T cell epitope diversity and clinical associations.

Allele	Spike					Membrane			Nucleocapsid						
	Lost	Decreased Predicted Binding	Gained	Increased Predicted binding	Lost	Decreased Predicted Binding	Gained	Increased Predicted Binding	Lost	Decreased Predicted Binding	Gained	Increased Predicted Binding	*X_total_*	COVID-19 induced Clinical Association	Reference
HLA-A*01:01	9	29	7	16	0	0	0	0	0	2	0	0	-17	Severe infection in Russia	(41)
HLA-A*02:01	5	24	6	9	1	1	1	1	0	3	0	0	-17	Protected in Russia	(41)
HLA-A*02:03	6	32	4	11	0	0	2	0	1	3	0	0	-25		
HLA-A*02:06	2	49	7	13	1	0	0	1	0	3	0	0	-34		
HLA-A*03:01	11	28	5	18	1	0	0	0	2	1	1	1	-18	Protected in Russia	(41)
HLA-A*11:01	15	32	10	21	0	0	0	0	3	2	1	1	-19	Associated with autoimmune and severe disease	(42–44)
HLA-A*23:01	15	40	5	10	0	0	0	0	1	2	0	1	-42		
HLA-A*24:02	16	38	5	5	0	0	0	0	1	2	0	1	-46	Associated with autoimmune disease	(44)
HLA-A*26:01	22	55	5	20	2	0	1	0	0	2	0	0	-55		
HLA-A*30:01	19	43	10	10	0	0	0	0	4	5	2	1	-48		
HLA-A*30:02	16	53	8	12	2	1	0	0	0	3	0	0	-55	Infection in USA	(45)
HLA-A*31:01	24	17	6	21	1	0	0	0	1	2	4	1	-13		
HLA-A*32:01	21	66	10	19	2	2	1	0	0	2	0	1	-62		
HLA-A*33:01	20	23	5	4	1	0	0	0	1	2	1	1	-36		
HLA-A*68:01	13	33	6	14	0	0	0	0	1	1	1	1	-26		
HLA-A*68:02	11	52	8	46	2	0	0	0	1	1	0	0	-13	Associated with reduced risk of ICU admittance	(46)
HLA-B*07:02	6	26	4	9	0	0	0	0	5	1	1	0	-29	Associated with severe disease	(46)
HLA-B*08:01	11	38	6	13	0	2	1	0	1	1	1	0	-39	Associated with autoimmune disease	(44)
HLA-B*15:01	18	41	9	31	0	0	1	0	0	0	0	1	-17	Survival in Egypt*, Asymptomatic in USA	(47–49)
HLA-B*35:01	18	69	11	29	1	0	0	1	1	3	0	0	-37	Associated with Subacute thyroiditis	(50)
HLA-B*40:01	4	10	2	5	1	0	0	0	0	2	1	0	-12		
HLA-B*44:02	7	21	1	0	1	1	0	1	0	2	0	0	-29		
HLA-B*44:03	6	17	1	2	0	0	0	1	0	0	0	0	-18		
HLA-B*51:01	5	40	4	5	1	0	1	1	0	3	1	0	-40	Associated with severe disease	(43, 46)
HLA-B*53:01	8	47	8	14	0	0	0	0	0	0	1	0	-34		
HLA-B*57:01	10	22	4	14	1	1	0	0	0	0	0	0	-17		
HLA-B*58:01	11	28	3	10	1	1	0	0	1	3	0	0	-32	Associated with severe	(19)

Epitopes lost, gained, and altered in predicted binding affinity in 27 HLA class I alleles. Epitope values reflect the number of unique epitopes affected. **X**_**total**_ describes HLA predicted clinical severity for all SARS-CoV-2 VOCs and protein product, spike (S), membrane (M), and nucleocapsid (N) and was generated using Equation 2, with more negative values indicating greater predicted clinical severity.

*Survival noted only in HLA-B*15 alleles generally.

### 3.4 Frequencies of affected HLA alleles in B.1.1.7 S, BQ.1.1 S, BA.1.1 M, and BA.4 N

To estimate how much of the United States population was potentially affected by the unbalanced epitope loss in BA.1.1 M, BA.4 N, and BQ.1.1 S protein variants, we utilized the haplotype frequencies cited by the US National Bone Marrow Donor Program (**Table 3**) (37). BQ.1.1 S epitopes sustained loss in 16/16 *HLA-A* alleles (making up approximately 92.3% population in EUR, 69.2% of AFA, 74% of API, 83% of HIS) and in 10/11 *HLA-B* alleles (62% EUR, 43.5% AFA, 31.2% API, and 38.5% HIS). BQ.1.1 S epitope gain was seen in all 16 *HLA-A* and 8/11 *HLA-B* alleles (48% EUR, 36% AFA, 26.2% API, and 29% HIS), with HLA-B*44:02 and HLA-B*44:03 experiencing only lost epitopes.

B.1.1.7 S and BA.4 N epitopes sustained decreased predicted immunogenicity in all 27 *HLA* alleles analyzed (16/16 *HLA-A* = 92.3% EUR, 69.2% AFA, 74% API, and 83% HIS; 11/11 *HLA-B* = 67.6% EUR, 44.8% AFA, 39.2% API, and 39.8% HIS) although only a fraction of HLAs analyzed experienced increased binding affinity in both repertoires (8/16 *HLA-A* and 6/11 *HLA-B* alleles in B.1.1.7 S). Likewise, only six *HLA-A* alleles (27.1% EUR, 23.5% AFA, 27.7% API, and 26% HIS) and one *HLA-B* allele (18.5% EUR, 9.5% AFA, 8.9% APA, and 11.2% HIS) experienced increased predicted binding in BA.4 N. BA.1.1 M repertoires lost epitopes for 9/16 *HLA-A* alleles (55.3% EUR, 39.3% AFA, 25.5% API, and 19.4% HIS), while only 4/16 *HLA-A* alleles experienced gains (35.7% EUR, 15.3% AFA, 17.8% API, and 2.9% HIS). Similarly, 6/11 *HLA-B* (29.2% EUR, 16% AFA, 27% API, and 19.4% HIS) lost epitopes, with only HLA-B*15:01 and HLA-B*08:01 gaining epitopes (19.1% EUR, 4.8% AFA, 5.1% API, and 7.3% HIS).

### 3.5 Predicted HLA clinical correlates of CD8^+^ T-cell epitope diversity

To summarize epitope difference between HLA and variant-specific S, M, and N, the number of unique epitopes experiencing loss, gain, and altered predicted binding was tabulated for the 16 HLA-A alleles and 11 HLA-B alleles analyzed (**Table 1**). The following equation was utilized to predict clinical severity of the 27 HLA haplotypes analyzed for individual protein products (Equation 1) and SARS-CoV-2 more broadly (Equation 2). Equation 2 was structured to reflect that clinical characteristics are affected by the net CD8^+^ T-cell epitope repertoire differences for all protein products.


(1) $$\begin{array}{l} X_{\left[\mathrm{HLA}\;\mathrm{allele}\right]\left[\mathrm{protein}\;\mathrm{product}\right]}\\ \;=n_{\;\left[\mathrm{HLA}\;\mathrm{allele}\right]\left[\mathrm{protein}\;\mathrm{product}\right]\mathrm{unique}\;\mathrm{epitopes}\;\mathrm{gained}}\\ \;+n_{\left[\mathrm{HLA}\;\mathrm{allele}\right]\left[\mathrm{protein}\;\mathrm{product}\right]\mathrm{unique}\;\mathrm{epitopes}\;\mathrm{increased}\;\mathrm{in}\;\mathrm{immunogenicity}\;}\\ \;-n_{\left[\mathrm{HLA}\;\mathrm{allele}\right]\left[\mathrm{protein}\;\mathrm{product}\right]\mathrm{unique}\;\mathrm{epitopes}\;\mathrm{lost}}\\ \;-n_{\left[\mathrm{HLA}\;\mathrm{allele}\right]\left[\mathrm{protein}\;\mathrm{product}\right]\mathrm{unique}\;\mathrm{epitopes}\;\mathrm{decreased}\;\mathrm{in}\;\mathrm{immunogenicity}} \end{array}$$

Equation 1: Predicted clinical severity, X, of an HLA allele specific for a SARS-CoV-2 protein product (spike, membrane, or nucleocapsid).


(2) $$X_{\mathrm{total}\left[\mathrm{HLA}\;\mathrm{allele}\right]}=X_{\left[\mathrm{HLA}\;\mathrm{allele}\right]S}+X_{\left[\mathrm{HLA}\;\mathrm{allele}\right]M}+X_{\left[\mathrm{HLA}\;\mathrm{allele}\right]N}$$

Equation 2: HLA predicted clinical severity for all SARS-CoV-2 VOCs and protein product, spike (S), membrane (M), and nucleocapsid (N).

Utilizing Equation 2, HLA-A*32:01, HLA-A*30:02, HLA-A*26:01, *HLA*-B*08:01, *HLA*-B*35:01, and HLA-B*51:01 were predicted to have worse clinical correlates when infected with SARS-CoV-2. Collectively, these six alleles are expected to affect approximately 7.0% of EUR, 9.1% of AFA, 5.3% of API, and 8.4% of HIS population for HLA-A and 22.8% of EUR, 12.5% of AFA, 12.2% of API, and 16.6% of HIS population for HLA-B alleles in the United States. Favorable clinical outcomes were predicted in HLA-A*01:01, HLA-A*02:01, HLA-A*31:01, HLA-A*68:02, HLA-B*15:01, HLA-B*40:01, HLA-B*44:03, and HLA-B*57:01 (**Tables 2**, **3**) (HLA-A: 50% EUR, 24.8% AFA, 17.8% API, and 33.4% HIS; HLA-B: 21.1% EUR, 8.2% AFA, 17.8% API, and 11.5% HIS). Our predicted clinical severity matched the reported clinical observations (42–48, 50), excluding HLA-A*11:01, which is explored further in the discussion (**Table 1**).

## 4 Discussion

Select studies have previously sequenced the HLA allele and viral epitopes of convalescent patients (16, 51, 52), but to our knowledge, none have explored the entire epitope repertoire of multiple SARS-CoV-2 variants with respect to the most common HLA allotypes. Although epitope screening has been conducted in cell lines (53, 54), no analysis of the COVID-19 peptidome exists on a population/epidemiological level. Therefore, our team utilized a computational approach aimed to model the immunogenic effects and clinical severity of SARS-CoV-2 variants in the most common MHC class I alleles comprising the United States population. Our bioinformatics analysis is consistent with the percentages of CD8^+^ epitope conservation (S: 87.6%–96.5%, M: 92.5%–99.6%, N: 94.6%–99%) found by Tarke et al. (15) (97%). As the virus mutated, an increasing proportion of spike epitopes experienced reduced predicted HLA binding, with 70% of Omicron BQ.1–XBB.1.5 S epitope repertoires experiencing decreased predicted HLA binding affinity (as compared with the roughly 3% and 15% affected in Delta AY.100–AY.44 and Omicron BA.1–BA.5 variants, respectively) (**Figures 1**, **2A**). The changes experienced by spike CD8^+^ epitopes highlight both the remarkable structural plasticity of the S protein and the selective pressures experienced by its gene, particularly following the widespread availability of vaccines in mid-2021 (**Figure 1**). Our findings suggest that viral genetic variation affecting CD8 T-cell epitope immunogenicity contributes to determining the clinical severity of acute COVID-19.

Our findings support the hypothesis that long-lasting immunity against SARS-CoV-2 variants will be difficult to achieve through vaccines based solely on the spike protein and using neutralizing antibodies as an efficacy endpoint. One strategy to achieve long-term immunity against COVID-19 is the development of T-cell vaccines (9, 55). When designing such vaccines, it is important that the epitopes selected are as invariant as possible and cover the maximum number of HLA haplotypes with even affinity distribution between HLA alleles (56). Our research identified several predicted epitopes that were gained and conserved between variants (**Figures 6**, **7**), including highly conserved nucleocapsid (*n* = 2) and membrane (*n* = 3) peptides predicted to elicit immune response through multiple HLA alleles (**Figure 7**). Additionally, the CD8+ T cell epitopes in this manuscript have been evidenced in previously published datasets (**Table 5**). To develop a pan-coronavirus vaccine, epitopes affecting conserved protein product regions should also be considered, such as AA 987–1205 in spike, AA 132–222 in membrane, and the AA 66–194 and 210–401 regions in nucleocapsid described in our findings. Lastly, considering that several HLA haplotypes, including HLA-A*11:01, HLA-A*24:02, and HLA-B*08:01, are associated with COVID-induced autoimmune disease (44), epitopes affecting these alleles must be carefully considered to minimize the risk of autoimmune adverse effects. *In-silico* and *in-vitro* experiments will be needed to confirm the bioinformatically predicted epitope gains and remove promiscuous peptides.

**Table 5:** HLA-I peptides confirmed in other peptidomic datasets.

Peptide	Parent Protein	Allele	Reference
HADQLTPTW	Spike	-A*24:02	(53)
TGSNVFQTR	Spike	-A*68:01	(54)
APRITFGGP	Nucleocapsid	-B*07:02	(54)

An alternative path to prevent or treat severe COVID-19 immunity is the development of personalized vaccines and/or treatment strategies. This requires the identification of haplotypes at risk of or protected from severe illness, which can be added to non-genetic risk factors to estimate the overall risk of severe outcomes. Our findings are significant because this study is one of the first to explore SARS-CoV-2 CD8^+^ epitope diversity in the context of HLA alleles found in most of the United States population. Our predicted clinical severity, ***X_total_*** (Equation 2), is consistent with previously published findings (**Tables 2**–**4**, **6**, **7**) and identified several novel candidate haplotypes that may be susceptible to severe disease, notably HLA-A*32:01, HLA-A*26:01, and HLA-B*53:01, and relatively protected from disease, such as *HLA*-A*01:01, HLA-A*31:01, HLA-B*40:01, HLA-B*44:03, and HLA-B*57:01 (**Tables 2**, **3**). All referenced clinical associations were consistent with our predicted estimates, except HLA-A*11:01, which was reported to have severe disease and COVID-induced autoimmune effects despite a low ***X_total_*** (−19), and HLA-A*01:01, which was reported to have severe infection in Russia despite a low ***X_total_*** (−17). The inconsistency of predicted/reported severity seen in HLA-A*11:01 may be explained through a combination of factors, including an association with COVID-induced autoimmune disease (42–44) and limited availability of CD8^+^ hepatitis B epitopes, with some reports (66) suggesting that chronic hepatitis B patients with this allele had less than 10% of known HBV epitopes. Therefore, with these findings being considered (66–68), HLA-A*11:01 patients with chronic, untreated, or poorly managed hepatitis B co-infection may be at greater risk of experiencing severe COVID-19 infection, even if the allele alone may not confer an increased risk of clinical severity. It is also important to be mindful of the considerable diversity generated from HLA polymorphism. A patient heterozygous for both *HLA-A* and *HLA-B* loci would have to account for the predicted clinical severity, ***X_total_***, of all four haplotypes to determine a true net predicted effect (not including the other MHC class I loci, -C). Therefore, clinical studies will be needed to confirm these findings. We hope that our computation study will encourage groups with access to large numbers of peripheral blood mononuclear cells from COVID-19 patients, such as the RECOVER cohorts, to analyze SARS-CoV-2 peptidomes in association with HLA haplotypes.

**Table 6:** Global summary of HLA Class I allele associated with severe COVID-19 infection.

Allele	Analysis	Unadjusted (95% CI)	Unadj. *p*-value	Adjusted (95% CI)	Adjusted *p*-value	Study size	Study location	COVID-19-induced clinical association	Reference
HLA-A*01:01	Principal component analysis		1.5 × 10^−4^			539	Russia	5/8 deceased patients homozygous for allele	(41)
HLA-A*03	Odds ratio		0.047			3,958	Spain	Not significant after corrections	(57)
HLA-A*11	Odds ratio			7.693 (1.06–55.6)	0.04	3,958	Spain	*After controlling for sequential organ failure assessment (SOFA)	(57)
HLA-A*11	Odds ratio	3.8 (1.4–10.3)	0.004	3.7 (1.5–9.2)	0.001	200	Iran		(58)
HLA-A*11:01:01:01	Odds ratio			2.26 (1.27–3.91)	0.013	613	Japan		(42)
HLA-A*23:01	Odds ratio		0.002	>2.5 (2.7–220.6)	0.038	801	Sardinia (Italy)	Exclusively present in moderate/severe disease	(59)
HLA-A*26	Odds ratio	3.04 (1.5–6.13)	0.0076			10,388	UK		(60)
HLA-A*30:02	Odds ratio			2.2 (1.4–3.6)	0.01	22,234	Midwest US	*Associated with African Americans	(45)
HLA-B*22	Odds ratio	1.66 (1.06–2.59)	0.002		0.032	4,376	Hong Kong		(51)
HLA-B*27	Odds ratio		0.045	4.63 (1.57–13.8)	0.005	578	Romania		(61)
HLA-B*27:07	Chi-squared with Yates + Bonferroni’s correction		0.00001		0.004	1,116	Italy		(19)
HLA-B*41	Chi-squared		0.05			69	Egypt		(47)
HLA-B*42	Chi-squared		0.01			69	Egypt		(47)
HLA-B*50	Odds ratio		0.007	7.94 (1.25–70.1)	0.037	578	Romania		(61)
HLA-B*51	ANOVA + Bonferroni correction				0.027	95	South Asia	More likely to be fatal than mild	(62)
HLA-B*52:01:01:02	Odds ratio			2.22 (1.22–3.87)	0.021	613	Japan		(42)
HLA-B*58:01	Chi-squared with Yates + Bonferroni’s correction		0.0131			1,116	Italy	Not significant after corrections	(19)
HLA-C*01	Odds ratio			11.182 (1.05–118)	0.04	3,958	Spain	*After controlling for sequential organ failure assessment (SOFA)	(57)
HLA-C*04:01	Odds ratio		0.02	5.4 (1.3–21.6)	0.07	22,234	Midwest US	*Associated with Hispanic Americans	(45)
HLA-C*04:01	Odds ratio			1.73 (1.20–2.49)	<0.021	299	Armenia		(63)
HLA-C*04:01:01:01	Odds ratio			11.01 (1.38–87.4)	0.02	96	India		(52)
HLA-C*05	Multivariate regression		4.7 × 10^−6^	*R*² = 0.37	0.00032		74 countries		(64)
HLA-C*12:02:02:01	Odds ratio			2.13 (1.18–3.71)	0.043	613	Japan		(42)
HLA-C*17	Chi-squared		0.03			69	Egypt		(47)
***Haplotype*** HLA-A*30:02, B*14:02, C*08:02	Odds ratio		5.9 × 10^−5^	10.3 (2.9–46.3)	.022	801	Sardinia (Italy)		(59)

OR, odds ratio; CI, confidence interval.

**Table 7:** Global summary of HLA Class I allele associated with low risk of or protection from COVID-19 infection.

Allele	Analysis	Unadjusted (95% CI)	*p*-value	Adjusted (95% CI)	Adjusted *p*-value	Study samples size	Study location	COVID-19-induced clinical association	Reference
HLA-A*02	Odds ratio		0.0156	0.57 (0.36–0.90)	0.0468	10,388	UK		(60)
HLA-A*02:01	Principal component analysis		0.0146			539	Russia		(41)
HLA-A*03:01	Principal component analysis		7.5 × 10^−^³			539	Russia		(41)
HLA-A*32	Odds ratio		0.004			3,958	Spain	Not significant after corrections	(57)
HLA-A*33	Odds ratio	0.11 (0.01–0.84)	0.010	0.03 (0–0.3)	0.006	578	Romania		(61)
HLA-B*12	Odds ratio	0.14 (0.02–1.01)	0.015			4,376	Hong Kong	Not significant after corrections	(51)
HLA-B*!5	Odds ratio	1,351.06 (4.5–405,445)	<0.001			69	Egypt		(47)
HLA-B*27	Odds ratio	0.34 (0.11–1.00)	0.047			4,376	Hong Kong	Not significant after corrections	(51)
HLA-B*44	Odds ratio		0.0069	0.45 (0.25–0.80)	0.0138	10,388	UK		(60)
HLA-B*35	ANOVA + Bonferroni correction		0.050			95	South Asia	More likely to be mild than fatal	(62)
HLA-C*05	Odds ratio		0.0101	0.36 (0.17–0.78)	0.0404	10,388	UK		(60)
HLA-C*06:02	Chi-squared with Yates + Bonferroni’s correction		0.0053			1,116	Italy	Not significant after corrections	(19)
HLA-C*15	Odds ratio	0.37 (0.28–0.92)	0.014	0.13 (0.03–0.53)	0.004	578	Romania		(61)

**Tables 6** and **7** heavily referenced **Table 1** from Hoeseinnezhat et al. (65).

CI, confidence interval.

## Data availability statement

The data presented in this study are deposited in the Figshare portal, figshare.com/s/e47f99c210177912283a, and github, github.com/elnaggarj/FoldX-PeptideDocking. All SARS-CoV-2 viral sequences generated by the LSUHSC Precision Medicine Laboratory were deposited into both GISAID and NCBI databases and are publicly available.

## Ethics statement

The viral sequences used for this study were obtained from nasopharyngeal swab samples collected by Ochsner Health clinics throughout Louisiana as part of routine medical care and retained as medical waste. Collection of these samples was authorized by the Ochsner IRB under protocol # 2021.221. Ochsner Health retained patient identifiers for medical waste under the State of Louisiana pandemic declaration, which mandated reporting of each COVID-19 case. However, no patient identifiers were used in this study. Fully de-identified samples were provided to the LSUHSC Precision Medicine laboratory. Results were analyzed by BIE and returned to Ochsner Health via a secure, HIPAA-compliant server. All samples are already publicly accessible in both GISAID and at the NCBI.

## Author contributions

GK: Conceptualization, Data curation, Formal Analysis, Funding acquisition, Investigation, Methodology, Project administration, Visualization, Writing – original draft, Writing – review & editing. JE: Formal Analysis, Methodology, Software, Visualization, Writing – original draft. MV: Formal Analysis, Investigation, Writing – review & editing. AF: Writing – review & editing, Data curation, Project administration. DT: Formal Analysis, Writing – review & editing. RR: Writing – review & editing, Formal Analysis. SL: Writing – review & editing, Formal Analysis. MS: Writing – review & editing, Validation. NN: Writing – review & editing, Validation. EG: Writing – review & editing, Formal Analysis. DF: Data curation, Writing – review & editing, Project administration. JC: Conceptualization, Data curation, Supervision, Writing – review & editing. LM: Conceptualization, Data curation, Funding acquisition, Supervision, Writing – review & editing.
