# Supplementary material for: A bioinformatic analysis of T-cell epitope diversity in SARS-CoV-2 variants: association with COVID-19 clinical severity in the United States population
Source: Front Immunol. 2024 May 9;15:1357731. doi: 10.3389/fimmu.2024.1357731 (PMC11112498; doi:10.3389/fimmu.2024.1357731)
Supplement: Supplementary file 1 [file DataSheet_1.docx]

Supplementary Material

# Supplementary Tables

## Supplementary Table 1

| **Supplemental Table 1** | | | | | |
| --- | --- | --- | --- | --- | --- |
| **Reference of HLA-B*15:01 structures utilized for peptide docking analysis** | | | | | |
| Structure Name | Deposit Date | Complex With | Organism | Expression System |  |
| 8ELG | 9/24/22 | SARS-CoV-2 Spike | Human | E. Coli BL21 (DE3) |  |
| 3C9N | 2/8/08 | SARS-CoV derived peptide | Human | E. Coli BL21 (DE3) |  |
| 6VB3 | 12/18/19 | Synthetic peptide | Human | E. Coli |  |
| 6UZP | 11/15/19 | Synthetic peptide | Human | E. Coli |  |
| 7XF3 | 3/31/22 | Influenza B virus | Human | E. Coli |  |
| 1XR8 | 10/14/04 | Human UbcH6 and Epstein-Barr Virus EBNA-3 | Human | E. Coli BL21 (DE3) |  |
| IXR9 | 10/14/04 | Human UbcH6 and Epstein-Barr Virus EBNA-3 | Human | E. Coli BL21 (DE3) |  |

*Note: Reference of crystalized HLA-B*15:01 structures extracted from the RCSB Protein Data Bank*

## Supplementary Table 2

For more information on Supplementary Material and for details on the different file types accepted, please see [here](https://www.frontiersin.org/guidelines/author-guidelines#supplementary-material).

| **Supplemental Table 2** | | | | | | | | | | | | |
| --- | --- | --- | --- | --- | --- | --- | --- | --- | --- | --- | --- | --- |
| **Complete List of Estimated ΔG for SARS-CoV-2 CD8+ peptides docked with HLA-B*15:01 by FOLDX** | | | | | | | | | | | | |
|  |  |  |  |  | ΔG (kcal/mol) | | | | | | |  |
| Peptide | Covid Strain | Mutation Type | Wuhan Predicted Binding | VOC Predicted Binding | 8elg | 7xf3 | 6vb3 | 6uzp | 3c9n | 1xr9 | 1xr8 | Notes (Altered from X to Y) |
| ALPFNDGVY | XBB.1.5 | Spike Increased Binding | 0.76 | 0.59 | -0.0996 | -0.5253 | 1.6263 | 0.2293 | 1.6068 | 0.6363 | -0.1897 | VLPFNDGVY to ALPFNDGVY |
| LERDLPQGF | XBB.1.5 | Spike Decreased Affinity | 0.12 | 0.82 | 0.4453 | 3.6447 | 2.8170 | 0.1648 | 3.2208 | 0.4052 | 0.1927 | LVRDLPQGF to LERDLPQGF |
| GQTGNIADY | XBB.1.5 | Spike Decreased Affinity | 0.08 | 0.18 | -2.0075 | 1.1077 | 0.7129 | 1.2210 | 1.1449 | 1.5290 | -0.1006 | GQTGKIADY to GQTGNIADY |
| HQPYRVVVL | XBB.1.5 | Spike Increased Binding | 0.59 | 0.56 | 0.9171 | -0.9554 | -0.1552 | 1.5139 | -3.0411 | 0.6024 | -0.4333 | YQPYRVVVL to HQPYRVVVL |
| LVKQLSSKF | XBB.1.5 | Spike Increased Binding | 0.06 | 0.04 | -1.1152 | -0.8479 | -1.2878 | -0.5966 | -0.8323 | -0.4570 | -0.3982 | LVKQLSSNF to LVKQLSSKF |
| CVADYSVIY | XBB.1.5 | Spike Increased Binding | 0.36 | 0.31 | 1.0302 | -0.2169 | 1.2148 | 1.0814 | 0.1826 | -0.2667 | 0.2702 | CVADYSVLY to CVADYSVIY |
| NCYSPLQSY | XBB.1.5 | Spike Increased Binding | 0.7 | 0.42 | 1.8250 | 1.4361 | 0.7030 | 1.7651 | 0.7594 | 1.3840 | 2.0799 | NCYFPLQSY to NCYSPLQSY |
| KLDDKGPNF | BA.1.1 | Nucleocap. Increased Binding | 1 | 0.5 | -0.5809 | 1.1021 | -3.2776 | -3.1637 | -1.7660 | -6.3115 | -3.0519 | KLDDKDPNF to KLDDKGPNF |

Note: Predicted binding values reflect predicted consensus percentile ranks generated from IEDB’s Tepitools, as described in the methods. Low scores correspond to high predicted binding affinities.

## Supplementary Table 3

| **Supplemental Table 3** | | | | | | | | | | | |
| --- | --- | --- | --- | --- | --- | --- | --- | --- | --- | --- | --- |
| **Complete List of Estimated binding energy for SARS-CoV-2 CD8+ peptides docked with HLA-B*15:01 by FOLDX** | | | | | | | | | | | |
|  |  |  |  | Binding energy (kcal/mol) | | | | | | |  |
| Peptide | Covid Strain | Mutation Type | VOC Predicted Binding | 8elg | 7xf3 | 6vb3 | 6uzp | 3c9n | 1xr9 | 1xr8 |  |
| CVADYSVLY | XBB.1.5 | Spike Gained | 0.36 | -1.63154 | 2.34666 | 1.81064 | -0.500407 | -0.598217 | 23.0922 | 2.19916 |  |
| YNSASFSTF | XBB.1.5 | Spike Gained | 0.96 | -3.72574 | -6.55485 | 0.978351 | 1.71327 | -2.75697 | 16.2357 | -2.92601 |  |
| ASFSTFKCY | XBB.1.5 | Spike Gained | 0.21 | -0.175898 | 0.240334 | -0.717659 | 0.131108 | 0.447979 | 0.632271 | -0.79669 |  |
| FQPTNGVGY | XBB.1.5 | Spike Gained | 0.12 | 1.41429 | -0.130615 | -3.3392 | -1.69677 | -7.62166 | 6.93107 | 3.57065 |  |
| YQPYRVVVL | XBB.1.5 | Spike Gained | 0.59 | -3.1373 | 1.03704 | -3.54689 | -2.94018 | 4.41745 | 12.0797 | -2.04342 |  |

*Note: Predicted binding values reflect predicted consensus percentile ranks generated from IEDB’s Tepitools, as described in the methods. Low scores correspond to high predicted binding affinities.*

# Supplementary Figures

## Supplementary Figure 1


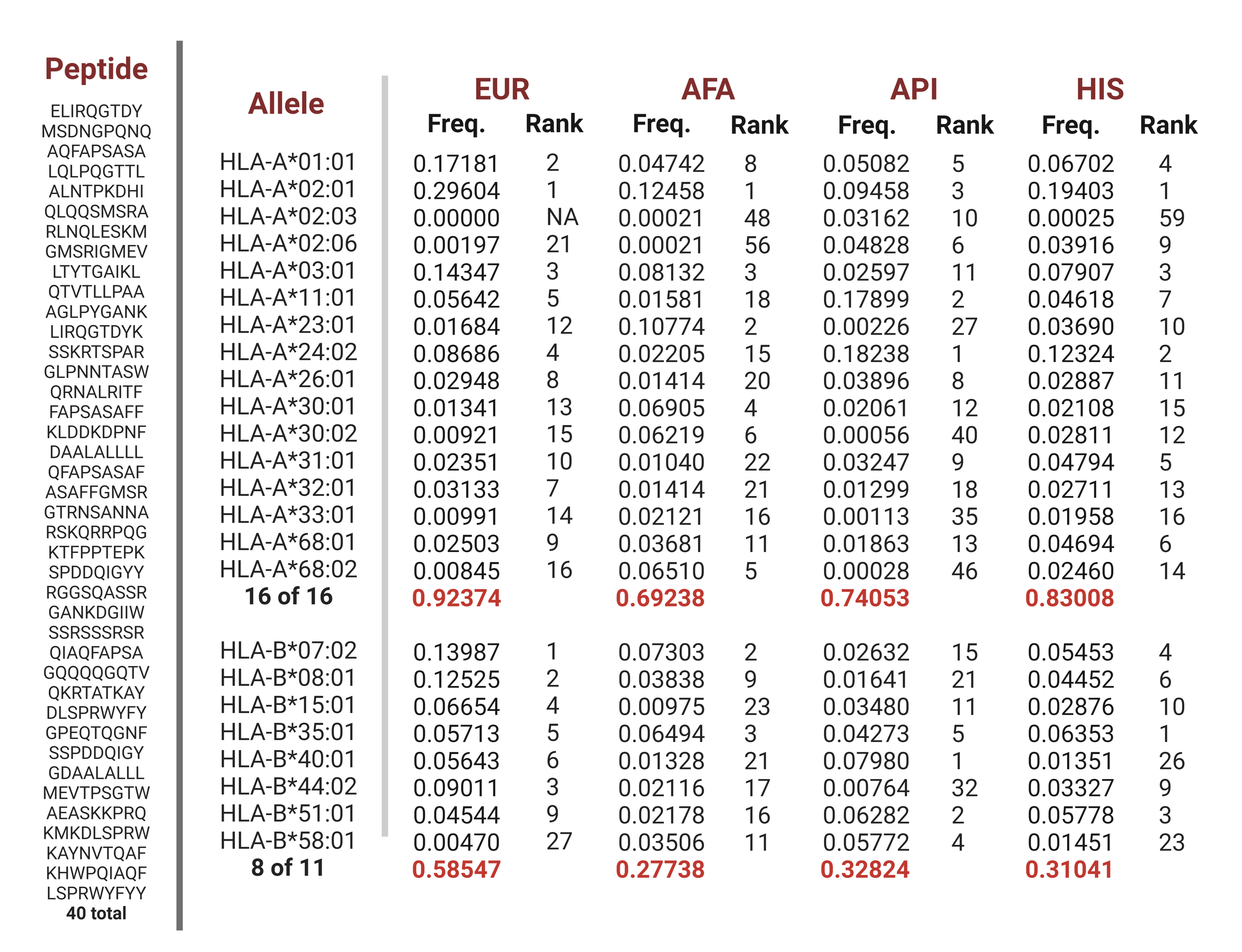


Supplemental Figure 1: BA.4 Nucleocapsid epitopes experienced a loss in immunogenicity, defined as a reduction in at least 10 positions in predicted percentile rank from IEDB generated concise ranking. Peptides marked with * indicates multiple alleles of the same peptide experienced decreased immunogenicity. HLA haplotype population frequencies and rank for Caucasians (EUR), African Americans (AFA), Asian (API), and Hispanic (His) populations were adapted from Gragert et al., 2013.


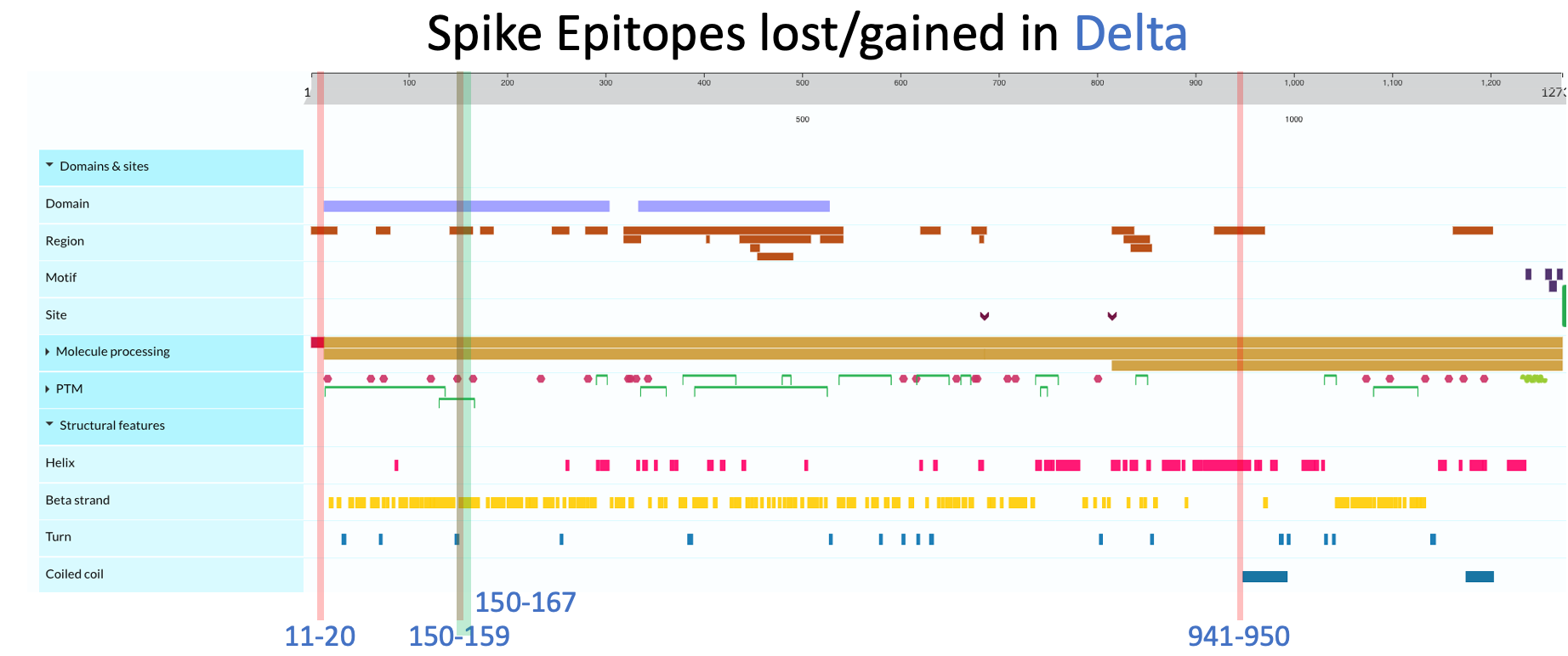


Supplemental Figure 2: Spike epitopes lost (indicated in red) and gained (green) in Delta variants. Protein characteristics were generated using UniProt’s Feature Viewer (6)

**
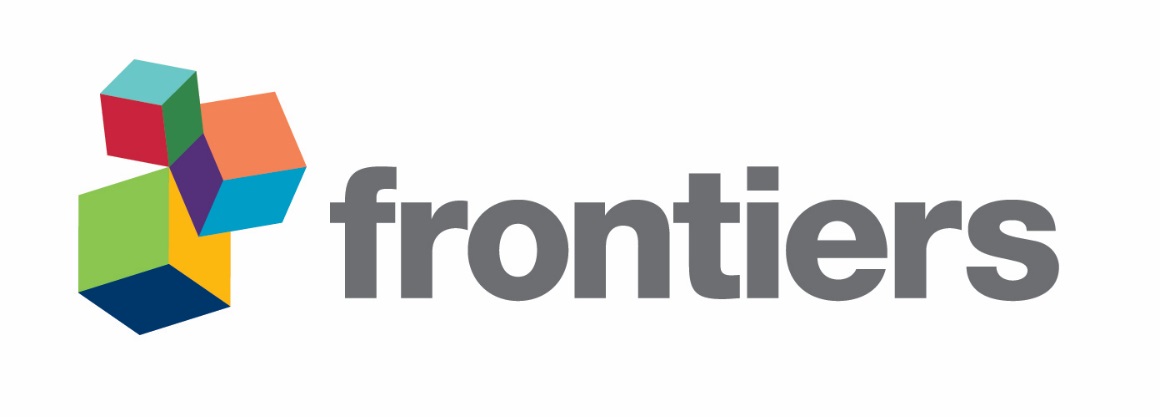
**
